# Malaria in travelers and local populations: Comprehensive study of incidence patterns and origin-based classification in Saudi Arabia

**DOI:** 10.1371/journal.pone.0335137

**Published:** 2025-10-17

**Authors:** Basmah Alharbi, Mawahib Ahmed

**Affiliations:** 1 Department of Basic Health Sciences, College of Applied Medical Sciences, Qassim University, Buraydah, Saudi Arabia; 2 Department of Medical Laboratories, College of Applied Medical Sciences, Qassim University, Buraydah, Saudi Arabia; Addis Ababa University, ETHIOPIA

## Abstract

**Background:**

Malaria continues to pose a significant public health threat in the Kingdom of Saudi Arabia (KSA), despite ongoing control efforts. Most malaria cases in the KSA are associated with travelers arriving from malaria-endemic regions. The rationale for studying malaria in the KSA stems from the country’s goal to eliminate the disease and address the increased risk of imported cases, which is heightened by substantial migration and religious tourism.

**Methods:**

This study aimed to assess the origins of malaria cases, the relative contribution of the different *Plasmodium* species involved, and the incidence rates across different age groups in the KSA. The Ministry of Health collected data on malaria cases in 13 administrative regions from January 2022 to December 2023. The chi-square test was used to analyze the data and determine the overall parameters and the rate of slide positivity.

**Results:**

The findings indicated that “imported” malaria cases were the predominant type of disease in the KSA. Out of 1,453,451 febrile cases examined, 0.7% (10,779) were positive across the 13 regions. In 2022, 688,629 cases were examined, with 0.9% (6,460) being positive. In 2023, 764,822 cases were examined, with 0.6% (4,319) being positive. Among these regions, Jazan exhibited the highest incidence rates (59%), followed by Makkah (20%), with a statistically significant difference (P = 0.046) between the regions. Malaria incidence was higher in patients aged ≥15 years. This study found significant variations (P = 0.002) in malaria incidence rates among different *Plasmodium* species. *Plasmodium falciparum* exhibited the highest rate at 63.5%, followed by *P. vivax*-*P. ovale* at 33%, *P. malariae* at 0.5%, and mixed infections where more than one species is involved at 3%.

**Conclusion:**

During the study period, imported malaria was the major type of malaria, especially in the Jazan region and Makkah. The highest incidence was caused by *P. falciparum*. These findings indicate the need for targeted interventions and public health strategies to mitigate the “imported” malaria burden, particularly among travelers.

## 1. Introduction

Malaria is a serious vector-borne illness that poses a significant threat to the global population. The alarming statistic that over 3 billion people are at risk of contracting malaria [1] stresses the urgent need for heightened awareness and the adoption of preventive measures to tackle this widespread health issue. According to the World Health Organization’s (WHO) 2024 report, 263 million malaria cases were confirmed globally in 2023, with international travel significantly facilitating the disease’s spread [2]. This data indicates a substantial increase, highlighting the ongoing challenges in the global fight against this devastating disease, which affects millions of people each year.

The continuously increasing malaria prevalence rate has severely affected human life, causing a significant number of deaths each year. The condition mostly occurs among people residing in tropical and subtropical regions, registering a massive percentage in the total mortality rate for malaria, as witnessed with the sustained threat the avoidable condition presents among these populations [1]. Optimal breeding conditions exist in tropical and humid climates, particularly in regions such as Africa and Southeast Asia, with mosquitoes serving as the primary vectors for the disease [3–5]. Individuals traveling from these areas may pose a risk of transmitting the infection to other regions across the world.

During the Hajj and Umrah seasons, the KSA welcomes over 4 million Muslim pilgrims from various parts of the world each year, which increases the likelihood of more malaria cases and may contribute to the spread of diseases through mosquito bites. To address this, the KSA has implemented proactive strategies to monitor and manage malaria cases, particularly among travelers from Yemen and other regions historically affected by the disease, including India and Sudan. Malaria is recognized as a significant cause of hospitalization for travelers from tropical and subtropical areas who exhibit fever symptoms in the KSA [6].

Malaria infections can be categorized into “indigenous,” “introduced,” and “imported” cases. “Indigenous” malaria pertains to instances contracted locally, devoid of any evidence suggesting importation or direct association with an “imported” case. “Introduced” malaria is defined as malaria contracted locally with substantial evidence directly associating it with a known “imported” case, signifying the initial local transmission [2,7,8]. “Imported” malaria refers to instances in which the infection was acquired outside the region where it was subsequently diagnosed.

According to Elagali et al. [9], malaria transmission dynamics in the KSA are characterized by climatic conditions. High temperatures with dryness are reported in summer, whereas cooler temperatures with high precipitation are reported in winter. This interplay of weather conditions shapes a distinctive environment that plays a crucial role in malaria transmission. In addition, higher numbers of cases were reported during times of religious festivities, attributed to the arrival of travelers in the country. As a result, the patterns and occurrences of this infectious disease vary across the country because of several epidemiological factors, such as climate variability, healthcare access, and human behavior, which are crucial for understanding malaria dynamics in the KSA. In response, the KSA has launched a comprehensive malaria control program designed to monitor and manage the disease’s incidence nationwide [10].

Most malaria infections in the southwestern regions of the KSA are caused by *P. falciparum*; however, *P. vivax* is more commonly found in the northwestern areas [11]. Understanding the distribution of these species is essential for developing effective malaria control and treatment strategies related to different geographic locations. *Anopheles arabiensis* has consistently been identified as the principal malaria vector in the KSA. Other species, including *An. dthali*, *An. sergentii*, and *An. stephensi*, have also been documented in entomological surveys and regional reviews, highlighting the diversity of potential vectors in the country [12–14]. Existing vector control strategies in the KSA, including insecticide-treated nets (ITNs), indoor residual spraying (IRS), and larval source management (LSM), have significantly impacted malaria dynamics by reducing mosquito populations and transmission rates. These methods, particularly ITNs and IRS, have been crucial in controlling the disease. Although several mosquito populations have developed resistance to pyrethroid insecticides, a wider range of insecticides can be used in IRS [15].

This situation emphasizes that malaria dynamics result from the interplay of environmental, biological, and socioeconomic factors. Moreover, it highlights the primary requirement for continuous scientific research and efficient prevention, in addition to expanding treatment provision to protect vulnerable populations. This study aimed to analyze the prevalence of malaria infections in the KSA over 2 years, focusing on the annual incidence rates across 13 administrative regions, diverse age demographics, specific parasites involved, and disease origins. To attain our objective, we conducted an epidemiological evaluation of the distribution of malaria cases in the KSA from January 2022 to December 2023. Gaining insight into the incidence patterns and origin-based classification of travelers and the local population in the KSA can significantly aid public health authorities in designing targeted interventions aimed at malaria elimination. The outcomes of this study will be valuable in enhancing the understanding of the origins of malaria and in informing strategies to address healthcare needs effectively.

## 2. Materials and methods

### 2.1 Study site

The KSA covers 2,217,949 square kilometers in Southwest Asia and is divided into 13 administrative regions. The country’s landscape features a diverse range of ecological and climatic conditions that affect the distribution of malaria vector species. Each region in the KSA has its own health department under the supervision of the Ministry of Health (MOH), which systematically records all malaria cases treated in public and private medical facilities. The standard method for detecting malaria parasites at the point of care in the country includes blood smear microscopy and rapid diagnostic tests (RDTs). Polymerase chain reaction (PCR) testing is also used when the parasite density is low or mixed infections are suspected, particularly in referral laboratories. In addition, *Plasmodium* species are commonly identified using PCR-based assays based on the 18S rRNA gene, a highly conserved gene among malaria parasites, which provides accurate identification of *P. falciparum*, **P. vivax*,* and other species [16].

### 2.2 Study design

This study used a retrospective observational cohort design, drawing on annual parasitological surveillance reports compiled by the Saudi MOH. The cohort included all confirmed malaria cases reported nationwide through the MOH surveillance system from January 2022 to December 2023. Cases were included if the surveillance records provided complete information on the parasite species, case origin, and classification. Reports missing essential demographic or clinical details such as unidentified species, incomplete classification, or duplicate entries were excluded.

The use of national surveillance data was justified by its comprehensive geographic coverage, standardized reporting procedures, and routine validation processes, which collectively ensured reliable documentation of malaria trends across the KSA and eliminated the need for additional ethical approval.

### 2.3 Statistical analysis

Statistical analysis for this study was conducted using SPSS (Version 28 – IBM) to examine trends and associations in malaria incidence across 13 regions of the KSA from January 2022 to December 2023. The chi-square test was used to investigate malaria incidence, positive cases, and slide positivity rates among the various regions. Furthermore, the chi-square test was used to evaluate the association between *Plasmodium* species types and geographic regions, and between disease origin classifications (“indigenous,” “imported,” and “introduced”) and the reporting year or region. Analysis of Variance (ANOVA) was used to analyze the differences in the prevalence means of *Plasmodium* species across the regions. Age-specific incidence rates were calculated and compared among the administrative regions. The level of statistical significance differences was considered at P < 0.05.

## 3. Results

Table 1 presents the annual incidence rates of malaria cases across the 13 administrative regions of the KSA. The table includes the number of examined cases, positive cases, and slide positivity rates, highlighting significant variations (P < 0.05) in malaria incidence among the regions. Makkah recorded 1,493 positive cases, which notably contributed to the national malaria burden. The Riyadh and Eastern regions also reported moderate incidence rates, whereas areas such as Qassim exhibited comparatively lower cases. Slide positivity rates were highest in regions with a greater number of cases, reflecting localized outbreaks or clusters of “imported” cases. The Jazan region showed the highest infection rate over the 2 years, followed by Makkah region, with statistically significant differences (P = 0.046) in malaria rates between the regions. Table 2 shows the analysis of *Plasmodium* species and the origin of malaria cases. The distribution shows that *P. falciparum* had the highest number of cases, establishing it as the dominant species. *P. vivax* and *P. ovale* were also prevalent, particularly in Makkah region, whereas mixed infections and *P. malariae* were rare. As shown in the table, no “indigenous” cases were reported in any region during the study period. All confirmed malaria infections were classified as either “imported” or “introduced,” with “imported” cases accounting for over 93% of the total.

**Table 1:** Annual distribution of malaria cases, slide positivity rates, and regional incidence across 13 Administrative Regions in the Kingdom of Saudi Arabia (2022–2023).

Region	2022		2023	
	Examined, n(%)	Positive, n(%)	Examined, n(%)	Positive, n(%)
Riyadh	33,423 (4.9)	220 (3.1)	20,324 (2.7)	172 (4.0)
Makkah	93,059 (13.5)	1,493 (23.1)	124,475 (16.2)	626 (14.5)
Medinah	43,708 (6.3)	244 (3.8)	51,094 (6.7)	135 (3.1)
Qassim	59,756 (8.7)	62 (1.0)	68,911 (9.0)	72 (1.6)
Eastern	140,574 (20.4)	368 (5.7)	221,027 (28.9)	322 (7.5)
Aseer	23,391 (3.4)	360 (5.6)	18,157 (2.4)	116 (2.7)
Tabouk	9,169 (1.3)	28 (0.5)	5,535 (0.7)	11 (0.3)
Ha`il	3,422 (0.5)	23 (0.4)	3,042 (0.4)	18 (0.4)
Northern	14,723 (2.1)	14 (0.2)	78 (0.01)	5 (0.1)
Jazan	190,815 (27.7)	3,579 (55.5)	171,098 (22.4)	2,774 (64.2)
Najran	30,438 (4.4)	37 (0.6)	26,477 (3.5)	50 (1.1)
Al-Bahah	6,532 (1.0)	31 (0.5)	7,579 (1.0)	11 (0.3)
Al-Jouf	39,619 (5.8)	1 (0.0)	47,025 (6.09)	7 (0.2)
Total	688,629 (100)	6,460 (100)	764,822 (100)	4,319 (100)

Regional differences in malaria incidence were analyzed using the χ² test; P = 0.046, indicating a statistically significant difference (*P < 0.05*).

**Table 2:** Distribution of malaria cases by *Plasmodium* species and disease origin classification across the Kingdom of Saudi Arabia in 2022.

Region	*Pf*, n(%)	*Pv-Po,* n(%)	*Pm,* n(%)	Mixed, n(%)	Indigenous, n(%)	Introduced, n(%)	Imported, n(%)
Riyadh	79 (2.1)	128 (5.4)	11 (30.6)	2 (0.7)	0 (0.0)	0 (0.0)	220 (3.6)
Makkah	522 (13.8)	899 (37.7)	16 (44.4)	56 (20.4)	0 (0.0)	103 (24.7)	1,390 (23.0)
Medinah	120 (3.2)	117 (4.9)	0 (0.0)	7 (2.6)	0 (0.0)	11 (2.7)	233 (3.9)
Qassim	19 (0.5)	40 (1.7)	2 (5.6)	1 (0.4)	0 (0.0)	0 (0.0)	62 (1.0)
Eastern	132 (3.5)	235 (9.8)	0 (0.0)	1 (0.4)	0 (0.0)	0 (0.0)	368 (6.1)
Aseer	246 (6.5)	107 (4.5)	4 (11.1)	3 (1.1)	0 (0.0)	70 (16.9)	290 (4.8)
Tabouk	27 (0.7)	1 (0.1)	0 (0.0)	0 (0.0)	0 (0.0)	0 (0.0)	28 (0.5)
Ha`il	10 (0.3)	10 (0.4)	1 (2.7)	2 (0.7)	0 (0.0)	0 (0.0)	23 (0.4)
Northern	3 (0.1)	11 (0.5)	0 (0.0)	0 (0.0)	0 (0.0)	0 (0.0)	14 (0.2)
Jazan	2,582 (68.5)	793 (33.2)	2 (5.6)	202 (73.7)	0 (0.0)	231 (55.7)	3,348 (55.39)
Najran	19 (0.5)	18 (0.8)	0 (0.0)	0 (0.0)	0 (0.0)	0 (0.0)	37 (0.6)
Al-Bahah	10 (0.3)	21 (0.9)	0 (0.0)	0 (0.0)	0 (0.0)	0 (0.0)	31 (0.5)
Al-Jouf	0 (0.0)	1 (0.1)	0 (0.0)	0 (0.0)	0 (0.0)	0 (0.0)	1 (0.01)
Total	3,769 (100)	2,381 (100)	36 (100)	274 (100)	0 (0.0)	415 (100)	6,045 (100)

*Pf* = *Plasmodium falciparum*; *Pv–Po* = *P. vivax* + *P. ovale*; *Pm* = *P. malariae*. “Mixed infections” = co-infections with two or more *Plasmodium* species. “Indigenous” = local infection with no link to an imported case; “Introduced” = local infection epidemiologically linked to an imported case; “Imported” = infection acquired outside the region of diagnosis (Ministry of Health criteria). Regional and species-level differences were assessed using the χ² test; P **= **0.002, indicating statistically significant variation among *Plasmodium* species across regions (*P < 0.05*).

Table 3 presents a comprehensive annual breakdown of each parasite type, facilitating a detailed examination of their evolution over the years. *Plasmodium falciparum* cases gradually decreased, with 3,079 cases in 2023. Additionally, there was a difference of 1,202 cases of *P. vivax* and *P. ovale* between the 2 years. Mixed infections involving multiple parasite species show a downward trend in 2023.

**Table 3:** Distribution of malaria cases by *Plasmodium* species and disease origin classification across the Kingdom of Saudi Arabia in 2023.

Region	*Pf*, n(%)	*Pv-Po,* n(%)	*Pm,* n(%)	Mixed, n(%)	Indigenous, n(%)	Introduced, n(%)	Imported, n(%)
Riyadh	86 (2.8)	80 (6.8)	3 (13.6)	3 (7.7)	0 (0.0)	0 (0.0)	172 (4.2)
Makkah	293 (9.5)	304 (25.8)	11 (50.0)	18 (46.2)	0 (0.0)	0 (0.0)	626 (15.5)
Medinah	101 (3.3)	24 (2.0)	0 (0.0)	10 (25.6)	0 (0.0)	0 (0.0)	135 (3.3)
Qassim	21 (0.6)	50 (4.2)	1 (4.5)	0 (0.0)	0 (0.0)	0 (0.0)	72 (1.8)
Eastern	172 (5.6)	148 (12.6)	2 (9.2)	0 (0.0)	0 (0.0)	0 (0.0)	322 (8.0)
Aseer	73 (2.4)	43 (3.6)	0 (0.0)	0 (0.0)	0 (0.0)	14 (5.1)	102 (2.5)
Tabouk	11 (0.4)	0 (0.0)	0 (0.0)	0 (0.0)	0 (0.0)	0 (0.0)	11 (0.3)
Ha`il	7 (0.2)	11 (0.9)	0 (0.0)	0 (0.0)	0 (0.0)	0 (0.0)	18 (0.4)
Northern	5 (0.2)	0 (0.0)	0 (0.0)	0 (0.0)	0 (0.0)	0 (0.0)	5 (0.1)
Jazan	2,284 (74.2)	477 (40.5)	5 (22.7)	8 (20.5)	0 (0.0)	260 (94.9)	2,514 (62.2)
Najran	21 (0.6)	29 (2.5)	0 (0.0)	0 (0.0)	0 (0.0)	0 (0.0)	50 (1.2)
Al-Bahah	5 (0.2)	6 (0.5	0 (0.0)	0 (0.0)	0 (0.0)	0 (0.0)	11 (0.3)
Al-Jjouf	0 (0.0)	7 (0.6)	0 (0.0)	0 (0.0)	0 (0.0)	0 (0.0)	7 (0.2)
Total	3,079 (100)	1,179 (100)	22 (100)	39 (100)	0 (0.0)	274 (100)	4,045 (100)

*Pf* = *Plasmodium falciparum*; **Pv–Po* *= *P. vivax* + *P. ovale*; *Pm* = *P. malariae*. “Mixed infections” = co-infections with two or more *Plasmodium* species. “Indigenous” = local infection with no link to an imported case; “Introduced” = local infection linked to an imported case; “Imported” = infection acquired outside the region of diagnosis (Ministry of Health criteria). Species-specific regional variation was analyzed using the χ² test; P = 0.002, showing statistically significant differences among *Plasmodium* species (*P < 0.05*).

Table 4 presents the descriptive results of the ANOVA analysis for malaria parasite prevalence across the 13 regions in the KSA. There was significant variation in the prevalence of *P. falciparum* compared with other types across these regions, with a mean square of 20.86 and a p-value of 0.002.

**Table 4:** Analysis of Variance (ANOVA) for regional differences in malaria parasite prevalence across the Kingdom of Saudi Arabia (2022–2023).

*Plasmodium* species	Sum of Squares	df	Mean Square	F	p
*P. falciparum*	250.30	12	20.86	5.62	0.002
*P. vivax and P. ovale*	150.85	12	12.57	3.48	0.035
*P. malariae*	90.25	12	7.52	0.89	0.712
Mixed infection	170.60	12	14.22	2.75	0.048

ANOVA was used to examine regional differences in *Plasmodium* species prevalence across the KSA. Differences were considered statistically significant at *P < 0.05*.

Tables 5 and 6 present several patterns when analyzing the data by age group. A significant (P = 0.002) pattern in malaria distribution was observed among the different age groups. Notably, over 94% of cases occur in individuals aged 15 years or older. This trend has been consistent over the years, with adults accounting for 96 cases in 2023. Children under 5 years old represent a relatively small percentage, ranging from 0.7% to 1.4% each year. Similarly, the age group of 5– < 10 years shows low occurrence rates, accounting for 0.3%–1.9% of yearly cases. The 10– < 15 years category experienced variations but generally remained below 2% of the total cases during the study period.

**Table 5:** Age-specific distribution of malaria cases across the Kingdom of Saudi Arabia in 2022.

Region	Age group				
	<5, n(%)	5-10, n(%)	11-14, n(%)	≥15, n(%)	Total, n(%)
Riyadh	2 (2.2)	2 (2.8)	0 (0.0)	216 (3.5)	220 (3.1)
Makkah	34 (38.2)	33 (45.8)	26 (27.4)	1,400 (22.6)	1,493 (23.1)
Medinah	3 (3.4)	4 (5.5)	2 (2.0)	235 (3.7)	244 (3.8)
Qassim	0 (0.0)	3 (4.2)	0 (0.0)	59 (1.0)	62 (1.0)
Eastern	4 (4.5)	2 (2.8)	3 (3.2)	359 (5.8)	368 (5.7)
Aseer	9 (10.1)	3 (4.2)	3 (3.2)	345 (5.6)	360 (5.6)
Tabouk	1 (1.1)	0 (0.0)	0 (0.0)	27 (0.4)	28 (0.5)
Ha`il	0 (0.0)	1 (1.4)	0 (0.0)	22 (0.3)	23 (0.4)
Northern	0 (0.0)	0 (0.0)	0 (0.0)	14 (0.2)	14 (0.2)
Jazan	36 (40.5)	24 (33.3)	60 (63.2)	3,459 (55.8)	3,579 (55.5)
Najran	0 (0.0)	0 (0.0)	1 (1.0)	36 (0.6)	37 (0.6)
Al-Bahah	0 (0.0)	0 (0.0)	0 (0.0)	31 (0.5)	31 (0.5)
Al- Jouf	0 (0.0)	0 (0.0)	0 (0.0)	1 (0.0)	1 (0.0)
Total	89 (100)	72 (100)	95 (100)	6,204 (100)	6,460 (100)

*n* = number of malaria-positive cases; % = positivity rate. Age-specific differences in malaria distribution across regions were evaluated using the χ² test (*P = 0.002*), indicating a statistically significant variation in infection rates among age groups (*P < 0.05*).

**Table 6:** Age-specific distribution of malaria cases across the Kingdom of Saudi Arabia in 2023.

Region	Age group				
	<5, n(%)	5-10, n(%)	11-14, n(%)	≥15, n(%)	Total, n(%)
Riyadh	5 (8.3)	0 (0.0)	5 (6.2)	162 (4.0)	172 (4.0)
Makkah	7 (11.6)	11 (13.8)	6 (7.6)	602 (14.7)	626 (14.5)
Medinah	0 (0.0)	3 (3.8)	7 (8.9)	125 (3.0)	135 (3.1)
Qassim	1 (1.7)	1 (1.2)	0 (0.0)	70 (1.7)	72 (1.6)
Eastern	0 (0.0)	4 (5.0)	7 (8.9)	311 (7.6)	322 (7.5)
Aseer	0 (0.0)	0 (0.0)	6 (7.6)	110 (2.7)	116 (2.7)
Tabouk	1 (1.7)	0 (0.0)	0 (0.0)	10 (0.2)	11 (0.3)
Ha`il	0 (0.0)	0 (0.0)	0 (0.0)	18 (0.4)	18 (0.4)
Northern	0 (0.0)	0 (0.0)	0 (0.0)	5 (0.1)	5 (0.1)
Jazan	45 (75.0)	61 (76.2)	48 (60.8)	2,620 (63.9)	2,774 (64.2)
Najran	0 (0.0)	0 (0.0)	0 (0.0)	50 (1.2)	50 (1.1)
Al-Bahah	0 (0.0)	0 (0.0)	0 (0.0)	11 (0.2)	11 (0.3)
Al-Jouf	1 (1.7)	0 (0.0)	0 (0.0)	6 (0.1)	7 (0.2)
Total	60 (100)	80 (100)	79 (100)	4,100 (100)	4,319 (100)

*n* = number of malaria-positive cases; % = positivity rate. Age-specific differences in malaria distribution across regions were evaluated using the χ² test (*P = 0.002*), indicating a statistically significant variation in infection rates among age groups (*P < 0.05*).

## 4. Discussion

The study findings indicate that international travelers have been the primary source of malaria in the KSA from 2021 to 2023, with all reported infections associated with individuals arriving from malaria-endemic regions. This highlights the significant role of international travel in malaria transmission and provides clear evidence that malaria in the KSA during 2022–2023 was primarily related to travel. The absence of “indigenous” cases suggests the successful elimination of local transmission, whereas the demographic distribution reveals a higher prevalence among those aged ≥15, highlighting the risk posed by adult travelers, including labor migrants and Hajj and Umrah pilgrims. Notably, the major entry points, such as Jazan and Makkah, have the highest number of cases. Jazan is on the border with Yemen, an area recognized for its endemic malaria. A considerable number of immigrants enter the KSA daily, whether seeking employment or fulfilling religious duties; this crossing has notably increased the risks of imported infections from Yemen [17]. Makkah attracts pilgrims and expatriate workers, recording the second-highest incidence rates, emphasizing the impact of large-scale pilgrimages. These findings indicate the critical need for ongoing surveillance, stringent screening at points of entry, and enhanced public health initiatives aimed at travelers to mitigate the entry of malaria into the country.

Malaria transmission in the KSA is confined to Aseer and Jazan, where vector populations persist because of favorable climatic conditions. The highest malaria incidence rates were recorded in Jazan, with *P. falciparum* as the predominant species. Environmental factors such as irrigation systems, seasonal rainfall, and temperature fluctuations influence mosquito breeding, contributing to persistent transmission [18]. The results of this study indicate that Jazan recorded the highest malaria incidence rates among the regions examined. This observation is significant because it confirms the ongoing public health challenge posed by malaria in this area.

The findings are similar to those of an earlier study conducted by Al-Mekhlafi et al. [19], showing that their cross-sectional study, spanning from April 1, 2018, to January 31, 2019, reported a total of 1,124 positive malaria cases. In contrast, Elhassan et al. [17] reported that malaria cases reported in Jazan between 2000 and 2014 were indigenous malaria rather than imported malaria.

Our findings indicated that most of the recent cases in the KSA from 2022 to 2023 are “imported.” However, Alzahrani et al. [18] found that malaria is “indigenous” in Jazan. The environmental conditions in this area likely contribute to the growth of mosquitoes. The origins of malaria indicate that a significant portion of the cases come from external sources, emphasizing the role of international travel in the transmission of this disease [19,20]. Each traveler may unknowingly pose a risk of spreading malaria, illustrating how global journeys can facilitate the movement of this disease across borders and communities. The absence of “indigenous” malaria cases reflects the successful implementation of control measures by the Saudi authorities, particularly through the national malaria eradication project.

The Makkah region experienced the second-highest rates of malaria in both the study years. This situation may partly stem from the entry of travelers from various parts of the world, including regions where malaria is endemic, as they come to the city to fulfill their Islamic duties. Recently, the rise in malaria cases in the KSA appears to reflect travel patterns to malaria-endemic countries.

Research suggests that a substantial portion of “imported” malaria cases are associated with travelers returning from these areas, as well as expatriates and their families visiting friends and relatives in their home countries where malaria remains a concern [21]. The positive findings from the slide results appeared to correlate with regions with notably high incidence rates, indicating a meaningful relationship between the two. Malignant malaria, driven by the parasite *P. falciparum*, has consistently emerged as the most prevalent and alarming form of malaria in the KSA. Alfaleh et al. [22] reported malaria cases in the KSA from 2017 to 2021. Their findings revealed that 76% of malaria cases during this period were attributed to the *P. falciparum* parasite, emphasizing the dominance of this particular species in the country’s battle against the disease. Alghamdi et al. [23] reported the epidemiology of malaria among international travelers entering Jeddah as an entry point. The findings showed that *P. vivax* has a significantly higher incidence than *P. falciparum* among patients during the study period.

The results of this study indicate that malaria incidence is significantly higher among individuals aged ≥15 years. While global studies suggest that malaria mortality is highest among young children, particularly in sub-Saharan Africa [24], this study highlights that in the KSA, ≥ 15 individuals bear the greatest burden of malaria. Moreover, regions such as Makkah demonstrate cases across various age groups, indicating broader exposure and potential risk factors for travelers and visitors from diverse parts of the world [6]. This analysis highlights the importance of prevention and treatment in areas with high incidence. Additionally, a study by Oshagbemi et al. [25], based on data from countries in sub-Saharan Africa, indicated that individuals aged 12 and older have a notably higher rate of malaria and associated mortality. However, according to a recent study by Geo et al. [26], children aged 0–4 face the highest risk of malaria infection in sub-Saharan Africa, and this risk decreases with age. This is in line with the demographic profile of the KSA’s traveler population, which is largely composed of adult pilgrims and migrant workers [27]. Restrictions on Hajj and Umrah participation for children under 12 years, particularly in 2023, further reinforce the lower incidence in younger age groups.

“Imported” malaria cases present a unique challenge for disease control efforts. Although no “indigenous” cases were reported in this study, the presence of vectors in some regions raises concerns about potential transmission if infected individuals introduce the parasite into receptive areas [21]. The continuous influx of malaria cases necessitates robust surveillance and rapid response strategies to prevent localized outbreaks [18]. Effective malaria control requires a combination of vector control, improved case detection, and prompt treatment strategies [24]. Current malaria control measures in the KSA include surveillance and case detection, involving screening at ports of entry and active monitoring in high-risk areas. Vector control strategies include the use of ITNs, IRS, and LSM. Public health awareness campaigns are essential in educating travelers and residents on malaria prevention and early detection. Although the data for this study were collected retrospectively and following standardized MOH protocols for diagnosis and reporting, underreporting may be a potential limitation, particularly in rural areas. Treatment protocols emphasize prompt diagnosis and treatment using artemisinin-based combination therapies to reduce transmission risk [24]. The Malaria Elimination Program in the KSA has made significant progress in reducing malaria incidence [28]. However, it remains essential to focus on preventing “imported” cases among travelers. To tackle this issue, a series of coordinated measures is recommended, such as promoting collaboration across borders and establishing strong disease and vector surveillance systems at key locations in border regions. Implementing case detection methods and monitoring artemisinin resistance are essential for timely interventions against resistance challenges.

Moreover, even in areas where malaria incidence has been successfully reduced to zero, continuing to provide ongoing training for clinicians and healthcare providers is important. Sharing updated diagnostic and treatment guidelines alongside training will aid in quickly identifying “imported” or transfusion-related malaria cases, thereby supporting the goal of complete malaria elimination.

The limitations of this study include the absence of laboratory-level metrics, such as parasite density and PCR confirmation rates, as the dataset relies on surveillance reports without gender differentiation. Additionally, the inability to distinguish between *Pv* and *Po* (referred to as ‘*Pv*-*Po*’) complicates our understanding of the burden of each species. The study also lacks data on seasonal patterns and contributing countries. Future documentation of treatment outcomes will be crucial for assessing potential drug resistance.

## 5. Conclusion

This study concludes that “imported” cases of malaria represent a significant public health concern in the KSA. Jazan and Makkah reported the highest incidence rates, with *P. falciparum* being the most prevalent type. To sustain malaria control, increasing surveillance, strengthening vector control, and expanding public health education are crucial. Prioritizing cross-border surveillance helps monitor “imported” cases, and increasing awareness among travelers, especially pilgrims and migrant workers, can significantly reduce infection rates.

Future research should assess the genetic diversity and vector resistance of malaria parasites to create more effective interventions. By implementing these measures, the KSA can advance sustainable malaria prevention and control.
